# Changes in the Amphibian Antibody Repertoire are Correlated With Metamorphosis and not With Age or Size

**DOI:** 10.1155/1992/28568

**Published:** 1992

**Authors:** Ellen Hsu, Louis Du Pasquier

**Affiliations:** 1 Department of Biology New York University New York NY 10003 USA; 2 The Basel Institute for Immunology 487 Grenzacherstrasse Basel 4005 Switzerland

**Keywords:** Antibody repertoire, *Xenopus*, metamorphosis, amphibian

## Abstract

Tadpole and adult *Xenopus*, manipulated to be of comparable size, exhibited stagespecific antibody expression. The production of adult-type higher-affinity anti-DNP antibodies proved to be independent of the age and size of the individual and is concomitant with the completion of metamorphosis. The appearance of new antibody specificities at such a time suggests that their expression occurs with the cell turnover and renewal during a period of morphological changes.


*Developmental Immunology*, 1992, Vol. 2, pp. 1–6
Reprints available directly from the publisher
Photocopying permitted by license only

© 1992 Harwood Academic Publishers GmbH
Printed in the United Kingdom

### Changes in the Amphibian Antibody Repertoire are Correlated with Metamorphosis and not with Age or Size

ELLEN HSU† and LOUIS DU PASQUIER‡*

†*Department of Biology, New York University, New York NY 10003*
‡*The Basel Institute for Immunology, 487 Grenzacherstrasse, 4005 Basel, Switzerland*

Tadpole and adult *Xenopus*, manipulated to be of comparable size, exhibited stage-specific antibody expression. The production of adult-type higher-affinity anti-DNP antibodies proved to be independent of the age and size of the individual and is concomitant with the completion of metamorphosis. The appearance of new antibody specificities at such a time suggests that their expression occurs with the cell turnover and renewal during a period of morphological changes.

KEYWORDS: Antibody repertoire, *Xenopus*, metamorphosis, amphibian.

### INTRODUCTION

The great morphological transformation that occurs in amphibian metamorphosis is accompanied by the expression of new serum and intracellular proteins. Among the development-stage-specific proteins studied in the South African frog, *Xenopus laevis*, are the hemoglobins (Kobel and Wolf, 1983; Just et al., 1977), creatine kinases (Robert et al., 1991), myosin heavy chains (Radice and Malacinski, 1989), vitellogenin (Huber et al., 1979; May and Knowland, 1980), antibodies (Du Pasquier et al., 1979; Hsu and Du Pasquier, 1984) and major histocompatibility (MHC) class I and II antigens (Flajnik and Du Pasquier, 1988; Du Pasquier and Flajnik, 1988; Rollins-Smith and Blain, 1990). In *Xenopus*, age and growth in size can be uncoupled from development by the use of goitrogens such as thiourea (Gasche, 1946) or sodium perchlorate (Pflugfelder, 1959), which suppress metamorphosis and produce permanent larvae. While some markers of differentiation such as adult hemoglobin (Just et al., 1977; Widmer et al., 1983) and MHC class I antigens (Flajnik and Du Pasquier, 1988) will gradually appear in the erythrocytes of these individuals despite their larval appearance, others such as vitellogenin inducibility (Huber et al., 1979; May and Knowland, 1980; Kawahara et al., 1987) and MHC class II antigens (Rollins-Smith and Blain, 1990) do not appear unless the animals are allowed to metamorphose. The expression of proteins unaccompanied by morphological changes probably results from changes in resident larval cells with the passage of time, and expression contingent on metamorphic events probably result from a novel (adult-specific) cell lineage.

We have suggested that stage-specific antibodies (Du Pasquier et al., 1979; Hsu and Du Pasquier, 1984a, 1984b) would be an example of the latter case. A major differentiation event in B lymphocytes is the rearrangement of immunoglobulin (Ig) genes, an event for which the mechanism is similar in *Xenopus* and mice (Schwager et al., 1988). Since allelic exclusion also operates in *Xenopus* B cells (Du Pasquier and Hsu, 1975), the differentiated lymphocyte is a cell containing permanent alterations at the Ig loci and incapable of changing its antibody specificity. The *Xenopus* adult-type antibodies are therefore probably produced by a population of lymphocytes different from the larval pool. The larval character of the antibody repertoire is unchanged by prolonging larval life by months (Hsu and Du Pasquier, 1984), suggesting that the lymphocytes bearing adult-type specificities appear only after meta-

*Corresponding author.

morphosis. However, this was not directly demonstrated.

Previous studies were performed only in larval and in adults greatly disparate in size and age (> 1 year). In the following experiments, we address questions of the relationship of specificities expressed to cell population size, to time or morphological state, and to metamorphosis.

### RESULTS

### Antibody Response in Tadpoles and Adults of Similar Size

We have shown previously that tadpoles produce anti-DNP antibodies differing from those of the adults in isoelectricfocussing properties (Du Pasquier et al., 1979; Hsu and Du Pasquier, 1984) and in affinity (Hsu and Du Pasquier, 1984a, 1984b). Because the tadpoles used in these experiments were considerably smaller (ca. 100 times by weight) than the adult counterparts, it was possible that the larval antibody response actually indicated an absence of some low-frequency B-cell clones normally present in the larger B-cell pool of an adult. Therefore, we tried to obtain animals of different developmental stages but of comparable size. In the experiment shown in Fig. 1, all the animals involved were 6-month-old siblings. The tadpoles were kept in sodium perchlorate from 4 weeks of age to the end of the experiment; given optimal conditions, they grew to very large sizes (1.2 to 2.8 g compared to normally 0.2 to 0.5 g). In contrast, their nonperchlorate-treated siblings had metamorphosed at the normal time (8 to 12 weeks), and were kept under crowded conditions so that they remained rather small (1.8 to 2.9 g compared to 10 to 25 g at 1–2 years of age). The froglets used originated from one particular aquarium, where the animals for unknown reasons developed very slowly and only began metamorphic climax after 4 months; the animals selected for immunization were just at the end of metamorphosis (stages 63–65). Although the tadpole and adult weights were comparable, the recently metamorphosed froglets were smaller; however, the amount of antigen administered to all animals ranged from 10 to 42 $\mu$g per gram body weight, which is within the effective dose range determined (Hsu and Du Pasquier, 1984) for adults (5 to 20 $\mu$g) and tadpoles (10 to 50 $\mu$g).

Although the tadpoles and adults were of comparable size, the anti-DNP antibodies they produced were different in affinity, in the same manner as in previous experiments where the two stages were greatly discrepant in size (Hsu and Du Pasquier, 1984a, 1984b). That is, most of the tadpoles produced anti-DNP antibodies with affinities lower than adults (Fig. 1). The one exceptional tadpole was not the largest member of the group, so that production of higher-affinity antibodies is not directly correlated with the size of the animal and hence the number of lymphocytes it has accumulated.

Thus, the difference in anti-DNP antibodies observed previously (Hsu and Du Pasquier,

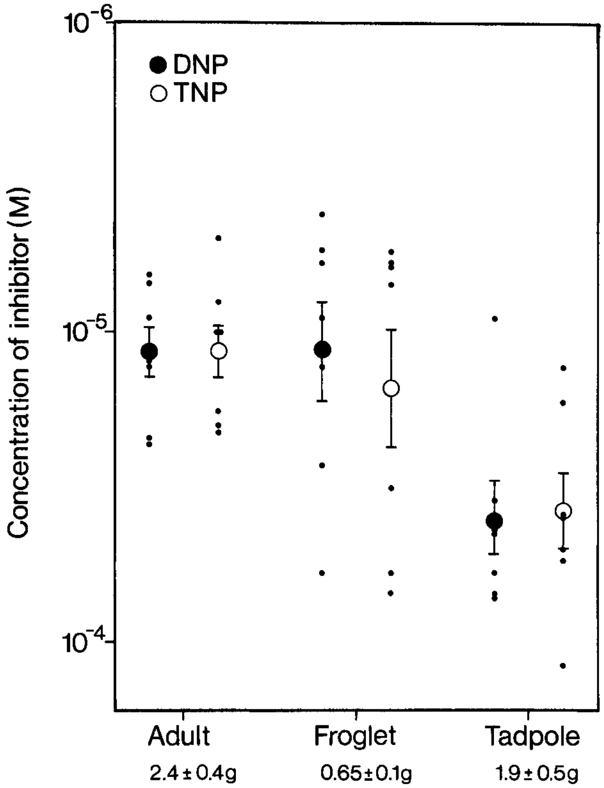

FIGURE 1. Comparison of anti-DNP antibody affinities from tadpoles and adults of similar size. Five-month-old sibling $LG_{15}$ tadpoles (stages 55–57, weighing 1.2–2.8 g, mean±S.D. was 1.9±0.5), metamorphosing tadpoles (stages 63–65, weighing 0.6–85 g, 0.65±0.1) and young adults (1.8–2.9 g, 2.4±0.4) were immunized with 5 $\mu$l of 5 mg/ml DNP/KLH in CFA and kept at 24–25°C for 3 weeks. The antisera were tested for inactivation and inhibition of inactivation of DNP-T4 phage. The data is presented as the geometric mean±standard error for the molar concentration of inhibitor (● DNP-lysin, ○ TNP-lysin) required for 50% inhibition.

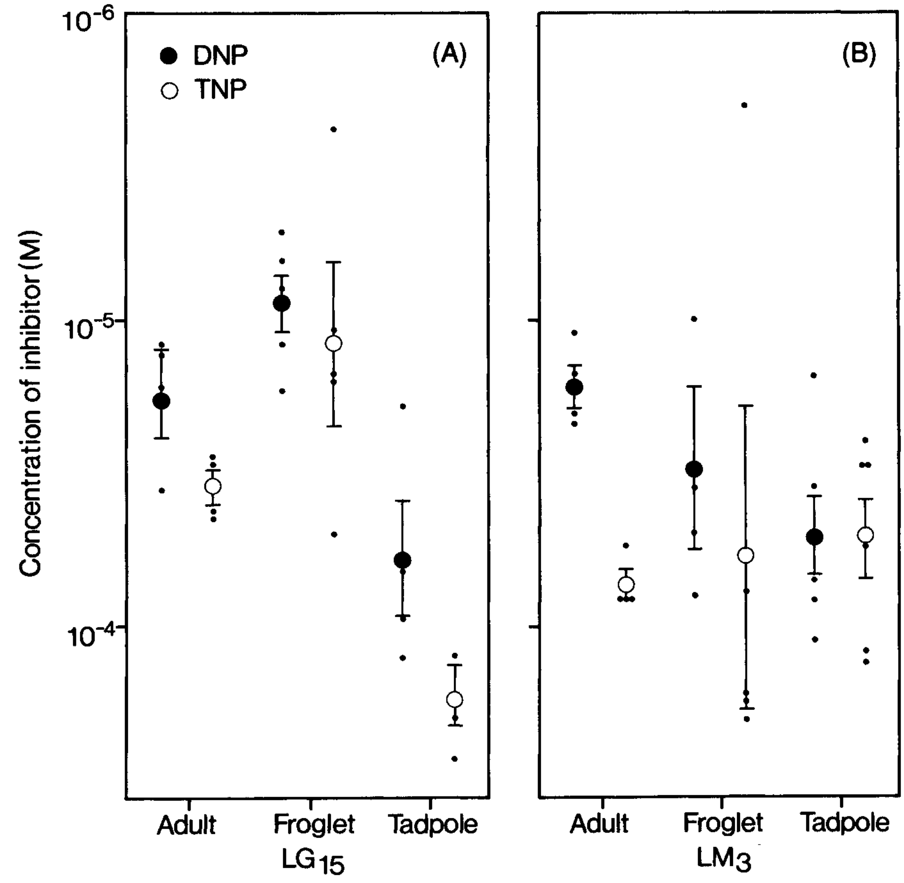

FIGURE 2. (A) Comparison of affinities of anti-DNP antibodies from larval and metamorphosed siblings. $LG_{15}$ tadpoles (stages 54–56) were injected with 5 $\mu$l of 2 mg/ml of alum precipitated DNP-KLH. Half of the group was placed in 0.1% sodium perchlorate to maintain the larval state and the other half was allowed to metamorphose over the 4-week immunization period. For comparison, 1-year-old $LG_{15}$ adults were injected with 50 ml of antigen. See legend to Fig. 1. (B) Comparison of affinities of LM3 tadpole and metamorphosing tadpole anti-DNP antibodies. Sibling tadpoles (stages 54–56) and metamorphosing tadpoles (stages 62–65) were immunized with 5 $\mu$l of 2 mg/ml of alum precipitated DNP-KLH; the tadpoles of earlier larval stages were kept in 0.1% sodium perchlorate after the immunization and the others metamorphosed. Two-year-old $LM_3$ adults were given 50 $\mu$l of the antigen; all animals were bled after 4 weeks and the antisera tested for DNP-T4 phage inactivation. See legend to Fig. 1.

1984) in tadpoles and adults, whose weight difference may have ranged from 10 to 100-fold, is not due to the low frequency of high-affinity anti-DNP clones, distinguishing by default animals by cell population size. Consistent with earlier observations (Hsu and Du Pasquier, 1984), tadpoles, maintained as larvae well beyond the time when metamorphosis would have occurred, make antibodies distinguishable from adults.

Most froglets produce antibodies of affinities overlapping with the adult range, although in this experiment they are about 30% the size of either tadpoles or adults. This observation supports our contention that the repertoire is a function of the developmental state and is probably not the result of cell accumulation.

### Antibodies Made by Larval and Metamorphosed Siblings

$LG_{15}$ tadpoles of one breeding were injected with DNP-KLH at stages 54–55. Half of this group was transferred into water containing 0.1% sodium perchlorate, and so remained in the larval state, while their siblings underwent metamorphosis over the 4-week immunization period. The antibodies produced by the froglets were higher in affinity than those made by their larval siblings (Fig. 2A). Although immunization had taken place when all the animals were tadpoles, the siblings that had gone through metamorphosis made different antibodies than if they had retained their larval state.

The adults immunized for this experiment produced anti-DNP antibodies of higher affinity than those of the tadpoles, generally overlapping with those of the froglets.

### Antibody Response in Animals Undergoing Metamorphic Climax

Sibling animals in metamorphic climax (stages 62–65) and of earlier developmental stages (54–56) were immunized with DNP-KLH. The latter were maintained in 0.1% sodium perchlorate, and up to sacrifice had not developed beyond stage 58. As in the previous experiments with the $LM_3$ and $LG_{15}$ *Xenopus* strains (Hsu and Du Pasquier, 1984a, 1984b), the tadpole antibodies to DNP were of lower affinity than those of the adults (Fig. 2B). In this instance, the adult antibodies clearly distinguished DNP from TNP, whereas those of the tadpole did not or were heteroclitic. In comparison, the range of affinities of the froglet antibodies overlapped with both tadpole and adult stages; sera of some individuals contained antibodies discriminating between hapten and analog, and another contained mostly heteroclitic antibodies.

In the experiment presented in Fig. 1, where animals were also immunized at the end of larval life, the affinities of the antibodies of froglets were also scattered over a range covering both tadpole and adult.

### DISCUSSION

When challenged with antigen, adults of *Xenopus* strain make antibodies of similar affinity and specificity (Du Pasquier and Wabl, 1977; Wabl and Du Pasquier, 1976). Antisera to DNP-KLH, sheep red blood cells, PC-KLH, when analyzed by isoelectricfocussing, showed sharing of, and identical, antibody bands among these genetically identical individuals (Du Pasquier and Wabl, 1978). This phenomenon is also found in isogenic tadpoles (Du Pasquier et al., 1979) but, in affinity and in IEF antibody bands, they differ considerably from adults of the same strain (Du Pasquier et al., 1979; Hsu and Du Pasquier, 1984). The larval antibody response is unaltered by the addition of adult T cells or by the reconstitution with adult T cells in the thymectomized tadpoles (Hsu and Du Pasquier, 1984a, 1984b). The sharing of IEF antibody bands among tadpoles of an isogenic strain suggests that the repertoire is peculiar to the larval state: some adult antibody bands never appear in tadpole antisera (Du Pasquier et al., 1979). When the larval period was greatly extended by the use of goitrogens, the antibody response was independent of both the elapsed time and the size of the tadpoles: large 7-month-old tadpoles still produced larval-type antibody bands (Hsu and Du Pasquier, 1984). Although these experiments suggested the existence of stage-specific repertoires, they could not rule out the possibility that there were not enough in the tiny tadpoles for low-frequency clones to be seen.

Here we have attempted to minimize the size differences by comparing runted adults with very large tadpoles. The results are clear, even very small metamorphosed animals produce antibodies with the characteristics of the adult developmental stage, whereas tadpoles of almost comparable size produce antibodies characteristic of the larval development stage. Even the froglet group, with an average body weight less than that of the tadpoles, tended to produce antibodies of affinities in the adult rather than the tadpole range, although, not surprisingly, there is a large degree of scatter. We suggest this scatter is indicative of an immune system in transition.

The change from the larval to the adult antibody repertoire occurs at metamorphosis. The production of higher-affinity anti-DNP antibodies, a characteristic of the adult $LM_3$ and $LG_{15}$ strains, only appears when metamorphosis has taken place; such antibodies do not appear when metamorphosis has been suppressed (Fig. 2A).

At the time of metamorphic climax, there is a great tissue turnover or replacement of almost all cell types, including those of hematopoietic lineages. Erythrocytes, for instance, are replaced by a cell population that synthesizes only adult hemoglobin and adult MHC class I antigens. These adult erythrocytes, moreover, differ in density and volume, as well as in general appearance (Flajnik and Du Pasquier, 1988). Our results make it likely that lymphocytes have a similar turnover. This is consistent with the previous suggestion of lymphocyte depletion, followed by rapid repopulation at the critical time (Du Pasquier and Weiss, 1973; Flajnik et al., 1987).

The tadpole is able to respond to DNP-KLH consistently by stage 52 (21 days of development), at which point there are about $10^4$ B cells (Du Pasquier and Weiss, 1973). If the average clone size 10, that is about three divisions per precursor, then the number of unique B-cell clones would be only $10^3$. Nevertheless, most tadpoles of a given strain manage to produce the same, or similar, antibodies to a given antigen. This would seem to require that the V-gene representation in animals of a given strain be "programmed" rather than "random," so that "favored" V genes will be consistently represented. These clones may be the source of B cells for the life of the tadpole, as the character of larval-type antibody response does not change, however, long larval life is artificially extended. New specificities appear only with the generation of the adult-type repertoire from the larger pool of new precursors that arise at metamorphosis.

A lack of functional junctional diversity has been observed in fetal and neonatal mouse $V_H$ sequences (Carlsson and Holmberg, 1990; Gu et al., 1990; Feeney, 1990; Meek, 1990) as well as in *Xenopus* (Schwager et al., 1991). Indeed, antibody diversity is limited at earlier stages of development in both mouse and *Xenopus*, but it is not clear whether this is related to the lack of junctional diversity. The larval-type immune system is an independent entity, not merely one in transition to a more "mature" adult version, so the imposition of a ceiling to antibody heterogeneity would only occur for greater functionality.

The nonrandom distribution of V genes expressed during ontogeny is well known in mammalian systems (for a review, see Alt et al., 1987; Wu et al., 1990). It is not clear why some $V_H$ genes are expressed earlier and at a higher frequency than others. In the tadpole, it is conceivable that a tiny larva, which must develop a functioning immune system in a short time with few cells, may require responsiveness to certain antigens earlier. There may have been selection for V-gene expression in order to increase the numbers of cells carrying important specificities. At this time, it is not clear which genes are preferentially expressed in the larval stages; sequences encoding 9 of the 11 described $V_H$ families (Hsu et al., 1989; Schwager et al., 1989; Haire et al., 1990) have been isolated from the tadpole B cell DNA and cDNA libraries (Schwager et al., 1991), and from tadpole mRNA hybridization data, most families appear to be expressed at the same levels in tadpoles and adults (E. Hsu and F.W. Alt, unpublished results).

### MATERIALS AND METHODS

### Animals

Clones of isogenic interspecies hybrids between *Xenopus laevis* and *Xenopus gilli* (LG) or *X. laevis* and *X. muellerei* (LM) were bred gynogenetically as previously described. The larval development stages (1–66) are described according to the normal table of Nieuwkoop and Faber (1967). Sodium perchlorate was used at a final concentration of 0.1% to block metamorphosis and prolong the larval life.

### Immunization

Adult LG or LM were given intraperitoneally (i.p.) 50–100 $\mu$l of 2 mg/ml dinitrophenylated keyhole limpet hemocyamin (DNP-KLH) emulsified in complete Freund's adjuvant (CFA) or precipitated with alum. Tadpoles and young metamorphs were injected with 5 $\mu$l of the antigen i.p. from a drawn glass syringe. The animals were all kept at 22°C, unless indicated, and bled after 4 weeks; blood was collected from the dorsal tarsus vein in adults and by cardiac puncture in froglets and tadpoles. Peritoneal fluid, which contains antibodies, was also withdrawn from tadpoles.

### Bacteriophage Inactivation Assay

The anti-DNP activity in the antisera was measured by a bacteriophage inactivation assay using DNP-coupled T4 phage. This technique, adapted from Adams (Adams et al., 1969), has been described fully in another publication (Du Pasquier et al., 1985). By the use of inhibitors (DNP-lysine) of the reaction, the relative affinities of the antisera can be determined; the relative specificity can be observed by using analogues of DNP (TNP-lysine). A range of six dilutions between $10^{-6}$ M and $3\times10^{-4}$ M inhibitor was used for all antisera. For each of the inhibition experiments, the antisera samples from all groups were tested together in order for the values to be comparable.

### ACKNOWLEDGMENTS

We wish to thank Ms. Anne Marcuz for her excellent technical assistance and Dr. Jean-Denis Franssen for help and encouragement. We also thank Dr. Charles Steinberg, for comments and criticisms, and Ms. Janette Millar, for secretarial assistance.

*(Received May 12, 1991)*

*(Accepted May 26, 1991)*

### REFERENCES

Adams M.H., Novik N., and Sila, M. (1969). Bacteriophages (New York: Wiley-Interscience).

Alt F.W., Blackwell K., and Yancopoulos, G.D. (1987). Development of the primary antibody repertoire. Science **238:** 1079–1087.

Carlsson L., and Holmberg (1990). Genetic basis of the neonate antibody repertoire: Germline V-gene expression and limited N region diversity. Internat. Immunol. **2:** 639–643.

Du Pasquier L., and Flajnik M.F. (1990). Expression of MHC class II antigens during *Xenopus* development. Dev. Immunol. **1:** 85–95.

Du Pasquier L., Blomberg B., and Bernard C.C.A. (1979). Ontogeny of immunity in amphibians: Changes in antibody repertoires and appearance of adult major histocompatibility antigens in *Xenopus*. Eur J. Immunol. **9:** 900–906.

Du Pasquier L., Flajnik M.F., and Hsu E. (1985). Methods used to study the immune system of *Xenopus (Amphibia, Anura)*. In Immunological Methods III, Lefkovits I. and Pernis B. Eds. (London: Academic Press), pp. 425–465.

Du Pasquier L., and Hsu E. (1983). Immunoglobulin expression in diploid and polyploid interspecies hybrids of *Xenopus:* Evidence for allelic exclusion. Eur. J. Immunol. **13:** 585–590.

Du Pasquier L., and Wabl M.R. (1977). The ontogenesis of lymphocyte diversity in Anuran amphibians. Cold Spring Harbor Symp. Quant. Biol. **41:** 771–779.

Du Pasquier L., and Wabl M.R. (1978). Antibody diversity in amphibians: Inheritance of isoelectric focusing in antibody patterns in isogenic frogs. Eur. J. Immunol. **8:** 428–433.

Du Pasquier L., and Weiss N. (1973). The thymus during the ontogeny of the toad *Xenopus:* Growth, membrane-bound immunoglobulins and mixed lymphocyte reaction. Eur. J. Immunol. **3:** 773–777.

Feeney A. (1990) Lack of N regions in fetal and neonatal mouse immunoglobulin V-D-J junctional sequences. J. Exp. Med. **172:** 1377–1390.

Flajnik M.F., and Du Pasquier L. (1988). MHC class I antigens as surface markers of adult erythrocytes during the metamorphosis of *Xenopus*. Dev. Biol. **128:** 198–206.

Flajnik M.F., Hsu E., Kaufman J.F., and Du Pasquier L. (1987). Changes in the immune system during metamorphosis of *Xenopus*. Immunol. Today **8:** 58–64.

Gasche P. (1946). Zur Frage des Angriffspunktes der Thiouracil. Experientia **2:** 24–26.

Gu H., Förster I., and Rajewsky K. (1990). Sequence homologies, *N* sequence insertion and $J_H$ gene utilization in $V_HDJ_H$ joining: Implications for the joining mechanism and ontogenic timing of Ly1 B cell and B-CLL progenitor generation. EMBO J. **9:** 2133–2140.

Haire R.N., Amemiya C.T., Suzuki D., and Litman G.W. (1990). Eleven distinct $V_H$ gene families and additional patterns of sequence variations suggest a high degree of immunoglobulin gene complexity in a lower vertebrate, *Xenopus laevis*. J. Exp. Med. **171:** 1721–1737.

Hsu E., and Du Pasquier L. (1984a). Ontogeny of the immune system in the *Xenopus* I. Larval immune responses. Differentiation **28:** 109–115.

Hsu E., and Du Pasquier L. (1984b). Ontogeny of the immune system in *Xenopus* II. Antibody repertoire differences between larval and adults. Differentiation **28:** 116–122.

Hsu E., Schwager J., and Alt F.W. (1989). Evolution of immunoglobulin genes: $V_H$ families in the amphibian *Xenopus*. Proc. Natl. Acad. Sci. USA **86:** 8010–8014.

Huber S., Ryffel G.V., and Weber R. (1979). Thyroid hormone induced competence for oestrogen-dependent vitellogenin synthesis in developing *Xenopus laevis* liver. Nature **278:** 65–67.

Just J.J., Schwager J., and Weber R. (1977). Hemoglobin transition in relation to metamorphosis in mammal and isogenic *Xenopus laevis*. Wilhelm Roux's Arch. Dev. Biol. **183:** 307–323.

Kawahara A., Kohara S., Sugimoto Y., and Amano M. (1987). A change of the hepatocyte population is responsible for the progressive increase of vitellogenin synthetic capacity at and after metamorphosis in *Xenopus laevis*. Dev. Biol. **122:** 139–145.

Kobel H.R., and Du Pasquier L. (1975). Production of large clones of histocompatible, fully identical clawed toads *(Xenopus)*. Immunogenetics **2:** 87–91.

Kobel H.R., and Wolff J. (1983). Two transitions of haemoglobin expression in *Xenopus:* From embryonic to larval and from larval to adult. Differentiation **24:** 24–26.

May F.E.B., and Knowland J. (1980). The role of thyroxine in the transition of vitellogenin synthesis from noninducibility to inducibility during metamorphosis in *Xenopus laevis*. Dev. Biol. **77:** 419–430.

Meek K. (1990). Analysis of junctional diversity during B lymphocyte development. Science **250:** 820–823.

Nieuwkoop P.D., and Faber J. (1967). Normal Table of *Xenopus laevis* (Amsterdam: North Holland).

Pflugfelder P. (1959). Beeinflussung der Thyreoidea und anderer Organe des Haushunes durch Kaliumperchlorat. Mit vergleichenden Untersuchungen an niederen Wirbeltieren. Roux' Arch. Entwickl. Mech. Org. **151:** 78–112.

Radice G.P., and Malacinski G.M. (1989). Expression of myosin heavy chain transcripts during *Xenopus laevis* development. Dev. Biol. **133:** 562–568.

Robert J., Du Pasquier L., and Kobel H.R. (1991). Differential expression of creatine kinase isozymes during development of *Xenopus laevis:* An unusual heterodimeric isozyme appears at metamorphosis. Differentiation **46:** 23–34.

Rollins-Smith L., and Blain P. (1990). Expression of class II major histocompatibility complex antigens on adult T cells in *Xenopus* is metamorphosis-dependent. Dev. Immunol. **1:** 97–104.

Schwager J., Bürckert N., Courtet M., and Du Pasquier L. (1989). Genetic basis of the antibody repertoire in *Xenopus:* Analysis of the $V_H$ diversity. EMBO J **8:** 2989–3001.

Schwager J., Bürckert N., Courtet M., and Du Pasquier L. (1991). The ontogeny of diversification at the immunoglobulin heavy chain locus in *Xenopus*. EMBO J. In press.

Schwager J., Grossberger D., and Du Pasquier L. (1988). Organization and rearrangement of immunoglobulin M genes in the amphibian *Xenopus*. EMBO J. **7:** 2409–2415.

Wabl M.R., and Du Pasquier L. (1976). Antibody patterns in genetically identical frogs. Nature **264:** 642–643.

Widmer H.J., Hosbach H., and Weber R. (1983). Globin gene expression in *Xenopus laevis:* Anemia induces precocious globin transition and appearance of adult erythroblasts during metamorphosis. Dev. Biol. **99:** 50–60.

Wu G.E., Atkinson M.-J., Ramsden D.A., and Paige C.J. (1990). $V_H$ gene repertoire. Seminars Immunol. **2:** 209–216.

